# Preventing Falls Among Older Adults Through Technology-Based Home Safety Assessment

**DOI:** 10.7759/cureus.89005

**Published:** 2025-07-29

**Authors:** Aya Hasegawa, Takayoshi Yamaga, Satoshi Tamaki, Chika Kimata, Tomoe Morita

**Affiliations:** 1 Department of Occupational Therapy, Faculty of Medical Science, Nagoya Aoi University, Aichi, JPN; 2 Department of Rehabilitation Medicine, Kamiiida Daiichi Hospital, Aichi, JPN

**Keywords:** fall prevention, home safety assessment, occupational therapy, older adults, online conferencing systems, remote assessment

## Abstract

Falls among older adults are a serious public health concern. In addition to exercise interventions, evaluating and modifying the home environment is essential for effective fall prevention. However, home assessments conducted via in-person visits are not frequently implemented due to constraints related to time and human resources. Recently, the use of Information and Communication Technology (ICT) in remote medical care has gained increasing attention, raising the possibility of its application in home evaluations. Nonetheless, there have been no prior reports evaluating the effectiveness of ICT-based home assessments as an alternative to traditional in-person visits. This report presents the case of an older adult female with bilateral knee osteoarthritis and mild cognitive impairments who had experienced recurrent falls. Concerned about her safety, the individual's family consulted an occupational therapist to conduct a home assessment. The Japanese version of the Westmead Home Safety Assessment was used for both in-person and online assessments. The results of both assessments were found to be highly consistent. After implementing countermeasures based on the hazards identified during the online assessment, the individual did not experience any falls over the subsequent six months. The individual’s family members reported high levels of satisfaction with the assessment process and its outcomes. This case suggests that online home assessments utilizing ICT may serve as a viable alternative or supplementary method to traditional home visits.

## Introduction

Falls are common among older adults, with approximately 30% of individuals aged 60 and older experiencing a fall each year [1]. In this report, a fall was defined according to the World Health Organization (WHO) as 'an event which results in a person coming to rest inadvertently on the ground or floor or other lower level'. These incidents may result in severe injuries, such as femoral neck fractures or head injuries, which may require hospitalization. Falls can adversely affect an individual's health and actions, such as reductions in physical and mental functioning as well as a loss of independence [2]. The occurrence of falls in older adults is intertwined with internal factors (including decreased physical and cognitive function) and external factors (including living conditions) [3]. Osteoarthritis of the knee is one of the conditions that increases the risk of falling [4]. Additionally, females are known to be at a higher risk of falling compared to males [5]. To address both internal and external risk factors, fall prevention guidelines strongly recommend exercise programs, home assessments, and modifications to the home environment [6]. In reality, at least one hazard is present in approximately 80% of homes inhabited by older adults [7]. Home evaluations and adjustments conducted by healthcare professionals using fall risk assessment tools are effective in reducing fall rates, especially among older adults at high risk [8].

Most home assessments and adjustments occur through home visits. However, specialized home assessments and adjustments require ample time and resources [9]. Although online evaluations may help increase adoption, there is limited evidence on the use of digital technologies in fall prevention [10]. To the best of our knowledge, there are no reports related to the use of home assessments conducted using online conferencing systems. Therefore, this study aimed to report a case that presented an opportunity to implement a remote home evaluation using an online conferencing system, in addition to a traditional in-person home visit.

## Case presentation

**Figure 1:**
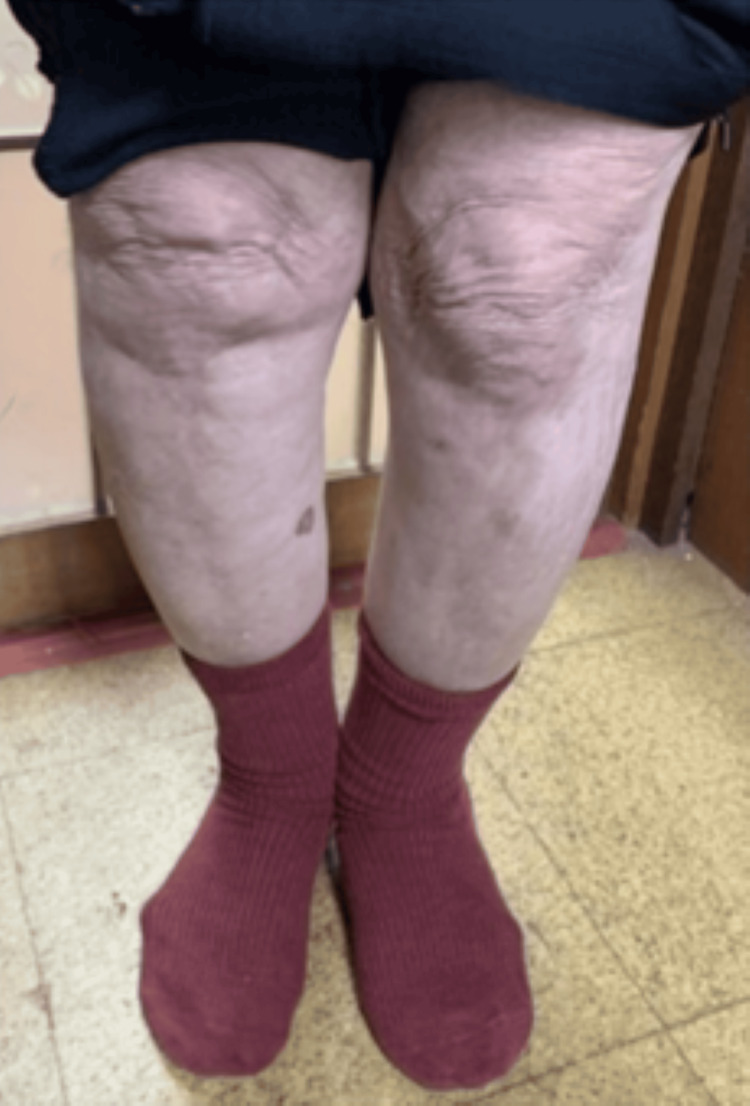
Knee deformity due to osteoarthritis

The individual was a female older adult in her early 80s. She had experienced bilateral knee pain for the past 14 years and was diagnosed with osteoarthritis (Figure 1). Eight years ago, she underwent percutaneous coronary intervention for angina pectoris and cataract surgery. One year later, she was diagnosed with Alzheimer's disease and initiated pharmacological treatment, including esomeprazole, clopidogrel, extended-release diltiazem, a rivastigmine transdermal patch, and memantine. Although some of these agents are known to potentially cause hypotension or dizziness, no such adverse effects were observed in this case. She remained able to walk independently both inside and outside the home and was independent in basic activities of daily living (ADL), such as eating and dressing. However, she began to require assistance with instrumental activities of daily living, including cooking and managing finances. Over time, she started experiencing falls at home, and two years ago, she sustained a right tibial plateau fracture from a fall. The fracture was managed non-surgically, and after one month of inpatient treatment, she was discharged and returned home.

Following discharge, the frequency of her falls increased compared to before the fracture, to approximately once monthly. Since the patient was living at home, detailed medical examination information was limited. Therefore, the specific cause of the fall risk could not be identified. Additionally, no recurrence of angina or cataracts has been observed. However, she appeared to pay no attention to the risk of falling. Her awareness of fall risk seemed low, and no behavioral changes were observed compared to her habits prior to the fracture. At times, she would even forget that she had fallen.

Her standing balance was assessed using the Berg Balance Scale (BBS) [11], a widely used tool for assessing fall risk in older adults. The scale scores range from 0 to 56 points. The risk of frequent falls increases with a BBS score of 45 or lower, and the risk becomes significantly higher at scores of 40 or below. Despite the individual scoring 50 on the BBS, she experienced recurrent falls. Her cognitive ability function was assessed using the Mini Mental State Examination-Japanese (MMSE-J) [12], a tool faithfully translated from the original MMSE and adapted to ensure cultural relevance for Japanese populations. The optimal cutoff score for identifying mild cognitive impairment and mild Alzheimer’s disease is 23/24; the individual scored 23 [12]. Points were deducted on items assessing time orientation, attention, recall, and visual construction. These findings suggest that the high frequency of falls, despite a high BBS score, may be attributable in part to attentional deficits and reduced risk awareness associated with cognitive decline.

Home visit-based environmental assessment

Post-discharge, the individual made use of daycare services twice a week, under the long-term care insurance system. During pickups and drop-offs from the daycare center, the care workers briefly checked the interior of the individual’s residence and asked her about any troubles she faced in daily life; however, no specialized assessments or modifications had been made to her home environment. Before the fracture, she experienced falls a few times per year. However, following discharge from a one-month hospitalization, the frequency of falls increased to approximately once per month and persisted at that rate for about two years. Concerned about this increase, her family members consulted staff at the day care center, which led to a home visit by an occupational therapist to assess her home environment, requiring over one hour of round-trip travel by car.

This home assessment was conducted using the Japanese version of the Westmead Home Safety Assessment (WeHSA-J), which is designed for fall prevention (Table 1) [13]. The WeHSA-J is a Japanese version of the Westmead Home Safety Assessment (WeHSA), developed with permission from the original author [14]. The tool has been partially modified to better suit Japanese housing structures and lifestyles, and it consists of 72 items in total. Furthermore, the WeHSA-J has been shown to have high inter-rater reliability and strong content validity. The WeHSA-J may only be applied following the completion of designated training. To ensure appropriate use of the WeHSA-J in this case, the occupational therapist participated in a four-hour training session conducted by the developers. Following this, they assessed older adults using WeHSA-J in the clinical setting, reported the results, and then received feedback. The assessment involved observing the individual as she performed her usual daily activities, both inside the home and in the surrounding outdoor areas. For each item on the WeHSA-J, if the individual was not using the relevant equipment or the equipment was not present, the item was marked as "not applicable." For applicable items, each was evaluated for the presence of fall hazards using the two-choice method. The family provided additional information when the case could not answer questions about their living situation.

**Table 1:** The Japanese version of the Westmead Home Safety Assessment (WeHSA-J) consists of 72 items. This table was created by the author, who developed the WeHSA-J. Reproduced with permission from Emeritus Professor Lindy Clemson, the original author of WeHSA [14].

Category (Number of items)	The types of hazards	
External trafficways (12)	Gates	Ice/snow no walkways
	Pathways/driveways	Lawns/gardens/grounds
	Steps/stairs	Doormat
	Ramps	Garage
	Steps/stairs handrails	Door opening
	Ramps handrails	Nightlighting
General/indoors (8)	Lighting	Telephone
	Tidiness/cleanliness	Heaters/fans
	Cleaning equipment	Commonly opened windows/curtains/shades
	Ironing area	Reaching for high places
Internal trafficways (10)	Floors ＆ floor covering	Ramps
	Floor mats	Stair/elevator approach
	Light switches/power points	Steps/stairs
	Space	Steps/stairs handrails
	Doorways	Ramps handrails
Mobility aid (1)	Mobility aid	
Pets (1)	Pets	
Living area (2)	Furniture	Lamps
Seating (1)	Seating	
Medication management (1)	Medication management	
Safety call system (1)	Safety call system	
Bedroom (6)	Bed	Bed lighting
	Wardrobes/cupboards	Bedside telephone
	Curtains/bed covers	Commode
Footwear (1)	Footwear	
Bathroom (6)	Location	Bath
	Dressing room	Bathroom/dressing room grab rails
	Bathroom	Bath grab rails
Toilet area (4)	Location	Toilet
	Floor covering	Toilet grab rails
Kitchen (14)	Proximity of kitchen to the eating area	Freezer
	Workplace	Oven
	Commonly used items	Grill
	Power points	Hot plates
	Sink	Microwave
	Jug/kettle	Dishwasher
	Fridge	Garbage
Laundry (4)	Location	Drier
	Washing machine	Clothes line

The occupational therapist who performed the home visit had 16 years of experience, having performed over 80 home evaluations and used the WeHSA-J more than 60 times. The occupational therapist was provided with documents regarding the individual’s basic information, a layout of the home, locations at which handrails were installed, and details regarding her fall history. Traveling to and from the individual’s residence took approximately one hour, and the assessment required approximately 45 minutes; overall, approximately two hours were required.

Home environmental assessment using online systems

Following the initial home visit and assessment, the individual’s family requested a follow-up for reconfirmation. However, due to limitations in time and staffing resources, an additional home visit could not be arranged. Therefore, a second occupational therapist conducted a home assessment using an online conferencing system via smartphone.

During the evaluation, the occupational therapist provided instructions on specific movements, while her family members recorded her condition using a smartphone. The assessments were conducted according to the WeHSA-J items. Therefore, information from the home visit assessment was not shared or discussed. The occupational therapist had been provided, beforehand, with the same information as the previous occupational therapist who conducted a home visit; however, the results of the previous WeHSA-J assessment were not made known to the second occupational therapist.

**Figure 2:**
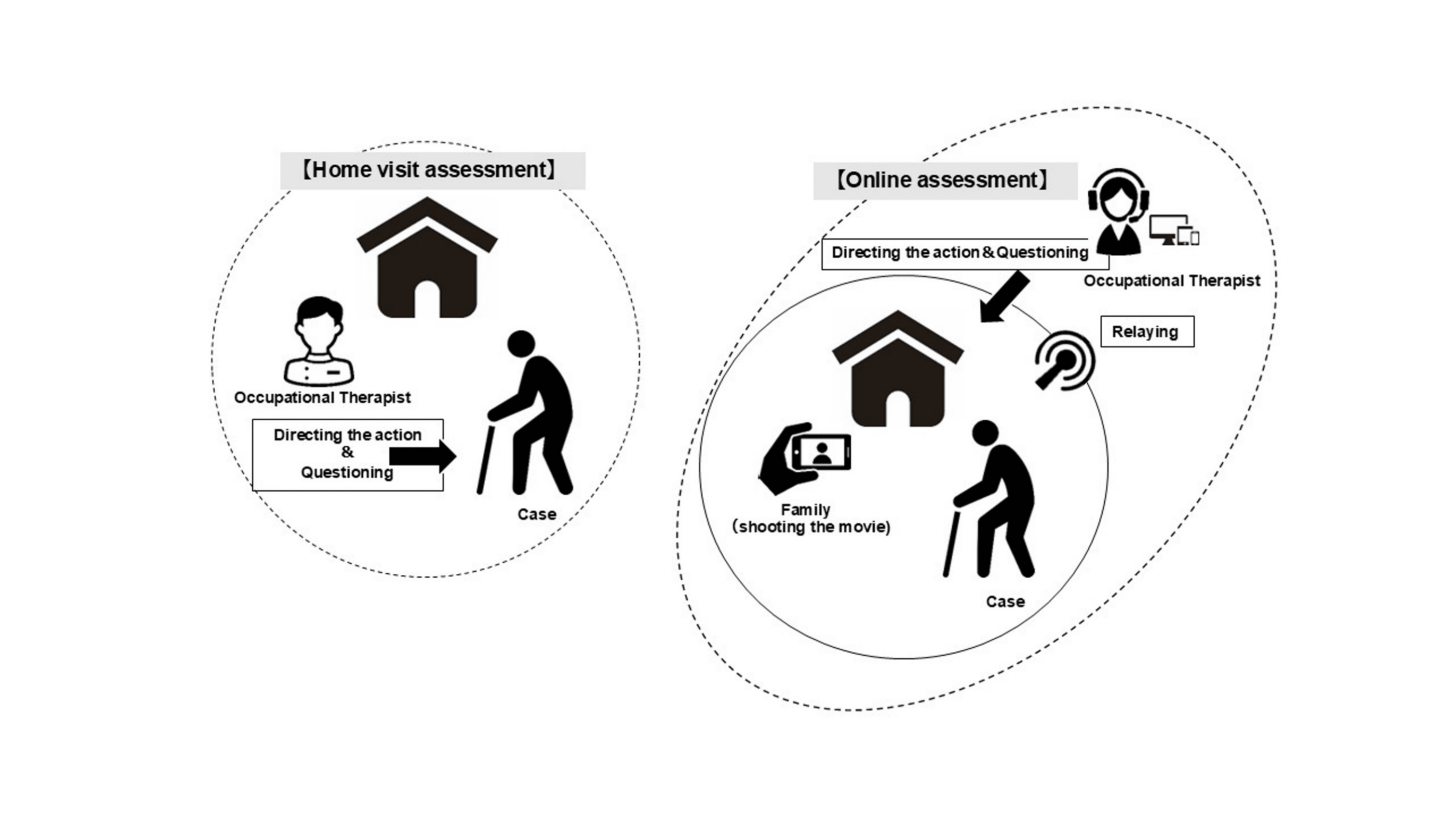
The assessment of home visit and online assessment for fall hazards This figure was created by the authors.

The occupational therapist conducted the assessment via the online conferencing system and had two years of experience. They had previously performed two home assessments and had used the WeHSA-J twice. It took approximately 15 minutes to establish the online connection, and the assessment itself required 45 minutes. Overall, the process took about one hour (Figure 2). Furthermore, the costs required to conduct the evaluation included the contract fees and communication charges for using the online conferencing systems. The occupational therapist utilized the online conferencing platform and telecommunications services with which they already had an active contract.

Outcomes

Out of 72 items on the WeHSA-J, 'not applicable' was checked for 20 items. The remaining 52 items were used to compare the results of the in-person home visit and the online assessment. During the home visit, 18 items were identified as hazardous, while 16 items were identified as dangerous during the online assessment. Overall, the results were consistent for 50 out of the 52 items (96.1%) (Table 2). The two items for which consistent results were not obtained were doorways and furniture (floor cushions). Both assessors discussed assessment results and evaluated fall prevention countermeasures for these two items. Therefore, it was determined that additional equipment was not required, as the individual would always hold on to doors and tables while entering and exiting rooms and when moving to the living room where floor cushions were placed, resulting in the risk of falls being low. All other equipment was installed using government-provided subsidies. No medical interventions for osteoarthritis, such as knee replacement surgery, intra-articular injections, or other medical therapies, were instituted at this time. Nonetheless, over the following five months, the individual did not experience any falls, and her family members expressed a sense of security and a high level of satisfaction. Her BBS score was 48 points, and ADL performance remained unchanged. Additionally, the family members would tidy the rooms and make preparations when others would visit the home. Although they noted some burden associated with recording the evaluation via the online conferencing system and the fatigue caused by occasional interruptions in network conditions, they felt that the method required less preparation, making them feel at ease.

**Table 2:** The agreement of Japanese version of the Westmead Home Safety Assessment (WeHSA-J) hazards between the online and home visit assessments

WeHSA-J item		Type of Hazard	Result		Agreement	Implemented measures on Online Assessment
			Online Assessment	Home Visit Assessment		
External trafficways						
	Pathways/driveways	Uneven/loose surfaces	Hazard	Hazard	Yes	Warnings about falling
	Steps/stairs	Steps too high/uneven heights （Back entrance, Entrance porch）	Hazard	Hazard	Yes	Install handrails
	External Steps/ramps handrails						Not present (Back entrance, Entrance porch)	Hazard	Hazard	Yes
	Lawns/gardens/grounds	Irregular ground surfaces	Hazard	Hazard	Yes		Warnings about falling
	Nightlighting	Steps/pathways not illuminated	Hazard	Hazard	Yes		
General/indoors						
	Lighting	Dark/dim	Hazard	Hazard	Yes	Install lighting
	Telephone	Access	Hazard	Hazard	Yes	Change the position of the telephone
Internal trafficways						
	Doorways	Other （The threshold of each rooms）	Hazard	No hazard	No	Install the triangular ramp on the threshold
	Internal Steps/stairs	Steps too high/uneven heights （Gest room)	Hazard	Hazard	Yes	Install handrails
	Internal Steps/stairs handrails	Not present (Gest room)	No hazard	Hazard	No	
Living area						
	Furniture	Other （Floor cushion）	Hazard	No hazard	No	Putting away the floor cushion
Bedroom						
	Wardrobes/cupboards	Access （Making and Storing a Futons）	Hazard	Hazard	Yes	Warnings about falling
Footwear						
	Slippers	Other （Easily slip off）	Hazard	Hazard	Yes	Change to suitable slippers
Bathroom						
	Bath	Poor access	Hazard	Hazard	Yes	Install bathroom and bath handrails
		Slippery bath				
	Bathroom grab rails	Not present	Hazard	Hazard	Yes	
	Bath grab rails	Not present	Hazard	Hazard	Yes	
Laundry						
	Location	Trafficway from laundry to drying area	Hazard	Hazard	Yes	Install handrail on the stairs to the 2nd floor
		Poor proximity to drying area (External, Internal)				
	Colthes line	Access （External，Internal)	Hazard	Hazard	Yes	
WeHSA-J：The Japanese version of the Westmead Home Safety Assessment						

## Discussion

To our knowledge, this is the first reported case suggesting that home assessments conducted via online conferencing systems may be effective in fall prevention. No previous studies have directly compared the results of in-person traditional evaluations with those conducted online, nor evaluated their effectiveness and applicability. This report offers new insights into potential future methods for conducting home assessments.

The most notable finding in this report is the high level of consistency (96.1%) between the results of the online assessment and the in-person home visit. The previous studies have demonstrated that home assessments conducted through in-person visits are effective in preventing falls among older adults at high risk [15]. Given the high degree of agreement between the two methods in identifying fall hazards, this case suggests that online home assessments may offer a comparable level of accuracy and effectiveness. In addition, previous studies have explored the use of photographs and videos for environmental assessments [16]. While the study did not compare these forms of media against the results of home assessments as traditionally conducted, an issue has been reported, in that necessary information was not obtainable depending on the angle at which recording or photography took place. The significance of assessing home environments of older adults, where the risk of falls is high, lies in conducting interviews related to movements and daily life in order for adults to gather information while making assessments regarding the suitability of individuals and home environments [17]. Photographs and videos do not allow for real-time evaluation and often fail to provide sufficient information. In contrast, online assessments allow for real-time evaluation of both the individual and the home environment. Because missing information can be supplemented during the assessment, this method is considered to be highly consistent with in-person evaluations.

Another important point highlighted in this report is the greater level of convenience of online assessments compared to home visit assessments. Firstly, this is because resources required in the form of time and human resources may be greatly reduced using this method. Home assessments conducted through in-person visits require substantial time and human resources, not only for the assessment itself but also for travel. In this case, the in-person assessment required approximately two hours, whereas the online assessment reduced the required time by 50%. Secondly, because smartphones can be used and no bulky equipment is necessary, online assessments can be conducted flexibly and at almost any time. In Japan, 80.5% of people own smartphones, and this number is increasing [18]. Most smartphones are equipped with built-in cameras, making them suitable for conducting online assessments. Furthermore, in terms of connectivity, the mobile communication infrastructure in Japan has significantly advanced, with major carriers providing widespread access to 4G and 5G services. These high-speed networks are used by the vast majority of the population and are available in most locations, supporting stable and accessible online communication [19]. Based on the two main findings presented above, online assessments appear to be a promising method for achieving both accuracy and efficiency.

Notably, among the two items where the in-person and online assessments produced inconsistent results, namely doorways and furniture (floor cushions), no falls occurred over the following six months, despite the only intervention being the provision of alerts. This suggests that, in this particular case, these two items may not have posed actual fall hazards. Furthermore, both were classified as “not hazardous” in the online assessment, that online assessments may have the potential to minimize the identification of non-critical hazards.

The limitations of this report lie in the low rates of internet usage in older adults [20]. Therefore, home environment assessments that rely on information and communication technology (ICT) may be difficult for older individuals to operate or fully understand, potentially limiting the widespread adoption of this method. However, the percentage of older adults who own smartphones has been increasing, and the introduction of ICT-based home assessments is expected to become easier in the future. Therefore, this issue is likely to be resolved over time. Furthermore, given that the case's cognitive impairment is part of a chronic and progressive condition, the potential for increased fall risk in the future cannot be excluded. Thus, the long-term sustainability of the observed fall prevention effect remains to be determined. As this report only provides details on one case study, further evaluation involving additional participants is necessary to determine whether similar results can be replicated.

## Conclusions

This case study demonstrates that home evaluations conducted via online conferencing systems may have an accuracy that is similar to that conducted via home visits. Such online assessments may be effective in identifying fall risks and implementing appropriate countermeasures. In settings where medical resources are limited, ICT-based assessment methods may serve as a practical and scalable option for fall prevention in the future.
